# Deletion of PRAK Mitigates the Mitochondria Function and Suppresses Insulin Signaling in C2C12 Myoblasts Exposed to High Glucose

**DOI:** 10.3389/fphar.2021.698714

**Published:** 2021-10-04

**Authors:** Ling Zhang, Jianguo Wang, Yu Tina Zhao, Patrycja Dubielecka, Gangjian Qin, Shougang Zhuang, Eugene Y Chin, Paul Y Liu, Ting C Zhao

**Affiliations:** ^1^ Department of Medicine, Rhode Island Hospital, Alpert Brown Medical School, Brown University, Providence, RI, United States; ^2^ Department of Surgery, Roger Williams Medical Center, Boston University School of Medicine, Boston, MA, United States; ^3^ University of Rochester School of Medicine and Dentistry, Rochester, NY, United States; ^4^ Department of Biomedical Engineering, University of Alabama at Birmingham, Birmingham, AL, United States; ^5^ Institute of Health Sciences, Chinese Academy of Sciences-Jiaotong University School of Medicine, Shanghai, China; ^6^ Department of Surgery and Department of Plastic Surgery, Rhode Island Hospital, Brown University, Providence, RI, United States

**Keywords:** C2C12 myoblasts, metabolic stress, mitochondria, high glucose, insulin signaling

## Abstract

**Background:** p38 regulated/activated protein kinase (PRAK) plays a crucial role in modulating cell death and survival. However, the role of PRAK in the regulation of metabolic stress remains unclear. We examined the effects of PRAK on cell survival and mitochondrial function in C2C12 myoblasts in response to high glucose stresses.

**Methods:** PRAK of C2C12 myoblasts was knocked out by using CRISPR/Cas-9 genome editing technology. Both wild type and PRAK^−/−^ C2C12 cells were exposed to high glucose at the concentration of 30 mmol/L to induce metabolic stress. The effect of irisin, an adipomyokine, on both wild type and PRAK^−/−^ cells was determined to explore its relationship with RPAK. Cell viability, ATP product, glucose uptake, mitochondrial damage, and insulin signaling were assessed.

**Results*:*** PRAK knockout decreased C2C12 viability in response to high glucose stress as evident by MTT assay in association with the reduction of ATP and glucose uptake. PRAK knockout enhanced apoptosis of C2C12 myoblasts in response to high glucose, consistent with an impairment in mitochondrial function, by decreasing mitochondrial membrane potential. PRAK knockout induced impairment of mitochondrial and cell damage were rescued by irisin. PRAK knockout caused decrease in phosphorylated PI3 kinase at Tyr 485, IRS-1 and AMPKα and but did not affect non-phosphorylated PI3 kinase, IRS-1 and AMPKα signaling. High glucose caused the further reduction of phosphorylated PI3 kinase, IRS-1 and AMPKα. Irisin treatment preserved phosphorylated PI3 kinase, IRS-1by rescuing PRAK in high glucose treatment.

**Conclusion:** Our finding indicates a pivotal role of PRAK in preserving cellular survival, mitochondrial function, and high glucose stress.

## Introduction

The mitogen-activated protein (MAP) kinase pathway has been identified to contribute critically to the regulation of metabolic stress and tissue injury in multiple tissues (Chen et al., 2010). Four distinct conventional MAPK pathways have been classified in mammals, which includes p38 MAPK, extracellular signal regulated kinases (ERKs) 1 and 2 (ERK1/2), c-Jun N-terminal kinases 1/2/3 (JNK1/2/3), and ERK5 (Roux and Blenis, 2004; Kyriakis and Avruch, 2012). We have previously demonstrated that activation of p38 and MAPKAP kinase-2 induced a preconditioning effect in response to myocardial ischemia and reperfusion injury (Zhao et al., 2001a; Zhao et al., 2001b; Zhao et al., 2013). p38 phosphorylation was closely related to protective effects in both pharmacological and agonist-induced preconditioning. There is evidence showing that metabolic stresses manifested an increase in the phosphorylation of p38, which is related to the insulin signaling pathway (Hemi et al., 2011; Ozcan et al., 2013; Jialal et al., 2016). p38 regulated/activated kinase (PRAK) is identified as a direct substrate of p38 MAPK. Other evidence indicates that PRAK is also activated by atypical MAPKs, ERK3/4, suggesting the involvement of PRAK/MK5 in both conventional and atypical MAPK mediated signal transduction pathways (New et al., 1998; Ni et al., 1998; Moens and Kostenko, 2013; Perander et al., 2016). PRAK was shown to phosphorylate FoxO1, FoxO3, and Rheb, the key component of regulation of insulin signaling pathways (Kress et al., 2011; Zheng et al., 2011; Chow et al., 2013). The study indicates that disruption of PRAK integrates a stress pathway with the mTORC1 pathway in response to energy depletion (Zheng et al., 2011). We have recently demonstrated that ablation of PRAK increased myocardial dysfunction and enhanced myocardial remodeling in myocardial infarction (Zhao et al., 2019). However, it remains unknown if PRAK is essential to modulating metabolic stress in response to high glucose stress in C2C12 cells.

Irisin is a recently identified proliferator-activated receptor-gamma coactivator-1α (PGCγ-1α)-dependent myokine, which is secreted by muscular tissues into circulation during exercise as a cleavage product of the extracellular portion of type I membrane protein fibronectin type III domain containing 5 (FNDC5) (Boström et al., 2012). Irisin was identified to play a key role in modulating insulin resistance and reducing oxidative stresses and apoptosis in different models (Lu et al., 2015; Park et al., 2015; Zhu et al., 2015; Jeremic et al., 2016). We have recently found that irisin preserves insulin signaling pathway in C2C12 myoblasts exposed to hyperglycemia, which acts through the PI3K pathway (Zhao et al., 2016; Wang et al., 2017; Yano et al., 2020). P38 was reported to activate PGCγ-1, upstream of irisin (Sanchis-Gomar and Perez-Quilis, 2014). Since PRAK serves as a direct downstream target of p38, it will be interesting to see whether there exists a link between PRAK and irisin to modulate a protective effect in C2C12 myoblasts. It remains to be established whether irisin could mitigate PRAK knockout induced cellular injury and mitochondrial dysfunction and rescue the insulin signaling pathway. In this study, we utilized CRISPR/Cas9 system to delete PRAK in C2C12 myoblasts, which were subjected to high glucose stress. We determine the effect of PRAK deletion on cellular viability, ATP synthesis, glucose uptake, mitochondria function, and insulin signaling in response high glucose stress. We have examined the function role of irisin, a newly identified hormone in preserving the mitochondrial function, increasing cellular viability and stimulating insulin signaling pathway in high glucose stress.

## Research Design and Methods

### Reagents and Antibodies

The MitoCapture detection kit was obtained from BioVision (Tokyo, Japan) for assessment of mitochondrial apoptosis. Recombinant human irisin was obtained from Cayman Chemical (Michigan, United States ). FCCP, the mitochondria oxidative and phosphorylation uncoupler, was obtained from Abcam (MA, United States ). Polyclonal rabbit active-caspase 3 antibody was purchased from Abcam. Other primary antibodies, including polyclonal rabbit β-actin, insulin receptor, phosphorylated AMPK, AMPK, phosphorylated IRS-1, and IRS-1 polyclonal rabbit antibodies, phosphorylated-PI3 Kinase (p85 (Tyr 485), PI3 Kinase, PRAK, and p38 antibodies were purchased from Cell Signaling Technology (Cell Signaling^Tm^, Beverly, MA). Phosphorylated PRAK (T182) antibody was obtained from Abcam (Waltham, MA). Irisin polyclonal rabbit antibody was purchased from Cayman Chemical (Ann Arbor, MI) 3-[4,5-dimethylthiazol-2-yl]-2,5-diphenyltetrazolium bromide (MTT) and 4,6-Diamidino-2-phenylindole (DAPI) were obtained from Life Technologies (Grand Island, NY).

### *In Vitro* C2C12 Myoblast Culture and Establishment of PRAK Knockout C2C12 Myoblasts

C2C12 skeletal myoblasts were purchased from American Type Culture Collection (ATCC, Manassas, VA). C2C12 myoblasts were grown in Dulbecco’s Modified Eagle Medium (DMEM), which was supplemented with 10% heat-inactivated fetal bovine serum (FBS) and 1% penicillin/streptomycin at 37°C in a humidified atmosphere of 5% CO_2_. pUC57sgRNA and Cas9 plasmids were obtained from Addgene (Watertown, MA). To achieve stable cell lines, myoblasts were transfected with plasmids encoding sgRNA/Cas9 and control plasmids by using Lipofectamine 2000 (Life Technologies, Grand Island, NY); these cell lines were designated as PRAK^−/−^ and wild type control in this study, respectively.

Stable transfection. A pair of PRAK oligo-DNAs consisting of 20 nucleotide-specific target sequences of PRAK was designed using the online tool http://crispr.mit.edu/(CRISPR Design of Massachusetts Institute of Technology). The sgRNAs of PRAK was synthesized by using the sequence including Forward: 5′-GTG​AGG​GTG​TTG​GCG​AGG​T-3′ and Reverse: 5′ ACC​TCG​CCA​ACA​CCC​TAC​AC-3′. The pUC57-T7-gRNA vector was then digested using BasI, and PRAK sgRNAs was cloned into the pUC57-T7-gRNA vector. Construction of the pUC57-PRAK-gRNA vector with DNA sequences above against PRAK was verified by sequencing. The plasmids including pUC57-PRAK-sgRNA and Cas9 nickkase expression vector were cotransfected in the indicated amounts into 5 × 10^5^ cells in 6-well plates using Lipofectamine 2000 Transfection Reagent (Invitrogen, Carlsbad, CA, United States), according to the manufacturer’s instructions.: 6 µg of pUC57-PRAK-sgRNA and 6 µg Cas9 nickase expression vector. The transfected C2C12 cells were trypsinized and resuspended to an approximately density of 10 cells/ml following 24 hours of transfection. Cell suspensions were aliquoted and seeded into a 96-well plate with a single cell per well. Positive C2C12 cells were screened by marking the single cells in the 96-well plate for an additional expansion of subcultures. The genome and protein extraction were carried out to determine the efficiency of PRAK knockout. qPCR was used to assess the knockout of PRAK in pUC57-PRAK-sgRNA positive and control cell lines using primers (Forward: 5-CAC​CCC​AGT​TTA​CCC​CTT​ACT-3; Reverse: 5-ACT​TCC​TGT​CAT​GAT​CTT​TTT​CCG-3) using an established protocol as previously described (Wang et al., 2017). Phenotypes of the transfected cells were also evaluated by immunoblotting.

### High Glucose Treatment Protocol

The high glucose treatment protocol is the same as described previously with modifications (Yano et al., 2020). Dulbecco’s modified Eagle’s medium (DMEM) supplemented with 1.0 mM sodium pyruvate and 4 mM of L-glutamine was used for culturing the cells. DMEM supplemented with low glucose (1,000 mg/L) and 1.0 mM of sodium pyruvate (Invitrogen) was used as a basic medium for C2C12 cell culture. The glucose concentration was induced at a concentration of 30 mmol/L while low glucose concentration was maintained at 5.6 mmol/L, which was chosen based on the methods in our previous study that used a concentration could effectively induce impairment in insulin signaling (Jeremic et al., 2016). After 48 hours of treatment, undifferentiated myoblasts will be subjected to measurements including molecular and cellular analyses as outlined below.

### Determination of Cell Viability

Cellular viability was assessed by measuring viability, which is based on the principle of reduction of 3-[4,5-dimethylthiazol-2-yl]-2,5-diphenyl tetrazolium bromide (MTT) (Sigma-Aldrich, St. Louis, MO) into blue formazan pigments in viable cells (Zhao et al., 2010; Du et al., 2015). Briefly, the culture medium was discarded at the end of experiments. The cells were then washed by using the buffer: 1 × PBS (PH 7.4. MTT (0.01 g/ml), then cells were dissolved in 1 × PBS, which was followed by adding MTT buffer and incubated at 37°C. Finally, cells were subjected to washing twice with the buffer containing 1X PBS and 1 ml of HCl isopropanol Triton (1% HCl in isopropanol; 0.1% Triton X-100; 50:1) with incubation for 5 min. The cell suspension was centrifuged at 16,000 g for 5 min. Optical density was measured using a spectrophotometer at a wavelength of 550 nm.

### Immunofluorescence Staining

Immunofluorescent staining was carried out as described before (Yano et al., 2020). Briefly, cells were washed with PBS at the end of each treatment, then fixed via immersion in 4% paraformaldehyde, which was followed by permeabilization in 1 × PBS buffer containing 0.1% Triton X-100 for 10 min. Cells were then washed with PBS buffer and blocked with PBS containing 1% BSA. They were incubated with polyclonal rabbit anti-active caspase 3 antibody (Abcam, Cambridge, MA) at a dilution of 1:200. Cells were then incubated with goat anti-rabbit Alexa Fluor 555 secondary antibody (Life Technologies) at a 1:200 dilution to develop fluorescent signals; 4′,6-diamidino-2-phenylindole (DAPI) was used to visualize the stained nuclei. The frequency of apoptotic positive cells was assessed in randomly selected areas and was normalized with stained nuclei.

### Mitochondrial Membrane Potential Assay

A reduction in mitochondrial membrane potential is an early indicator of apoptosis induction (Yano et al., 2020). The membrane potential was measured using the MitoCapture mitochondrial apoptosis kit (BioVision, Milpitas, CA, United States ) according to the instructions as described. Briefly, once the C2C12 myoblasts were subjected to the treatments as outlined in the protocol above, they were incubated in the cultured medium containing the MitoCapture reagent at a 1:1,000 dilution for 20 min at 37°C. Following two washes with PBS, fluorescent signals were measured using a confocal laser scanning microscopy (LSM 700, Carl Zeiss). The signal for capturing red fluorescence was excited to 530 nm and detected at 630 nm; the signal for capturing green fluorescence was excited to 488 nm and detected at 530 nm. The intensity for quantifying fluorescent signal was assessed by using NIH Image J software.

TMRM assay. Once C2C12 myoblasts were subjected to different treatment as outlined above, they were incubated in serum free medium containing TMRM at a concentration of 300 nM (Thermo Fisher) for 30 min at 37°C in the presence and absence of pre-treatment with FCCP (20 µM) for 20 min. The fluorescent signals were acquired at excitation to 547 nm and detection at 573 nm. Intensity of fluorescent signals was evaluated through NIH Image J imaging software.

### Measurement of ATP Contents

Protein concentrations of mitochondrial fractions were determined using Micro BCA Protein Assay Kit™ (Thermo-Fisher Scientific). ATP content was measured by ATP Colorimetric/Fluorometric Assay Kit™ (BioVision) according to the manufacturer’s instructions. Absorbances (OD 570 nm) were measured by the microplate photometer.

### Analysis of Glucose Uptake

The glucose uptake was measured as described as before (Yano et al., 2020). Briefly, culture medium was removed and phosphate-buffered saline (PBS) wash buffer was used to rinse cells and then continued to incubate at 37°C in buffer, in which 2-NBDG (200 μmol/L) was included into the medium. After 1 h of incubation, fresh incubation medium containing 2 mM glucose was added for an additional 5 min. The cells were then washed twice in PBS and incubated at room temperature in cell lysis buffer (1% sodium deoxycholate, 40 mM KCl, and 20 mM Tris [pH 7.4]) for 5 min. After this, cells were collected, homogenized, and centrifuged at 12,000 × g for 5 min at 4°C. The supernatants were collected to measure with a fluorescence microplate assay using a microplate read er. A standard curve graph was produced by measuring fluorescence of 2.5–20 mM 2-NBDG in lysis buffer to quantify 2-NBDG ([2-NBDG] uptake. The concentration of transported 2-NBDG was normalized to the amount of protein.

### Western Blotting

Protein levels were measured by western blotting using cell lysates (50 μg/lane) as described previously (Wang et al., 2017; Yano et al., 2020). In brief, the blots were incubated with their respective polyclonal antibodies, which included polyclonal rabbit phosphorylated IRS-1, IRS-1 phosphorylated AMPKα, AMPKα, phosphorylated-PI3 Kinase p85 (Tyr 485) and PI3 Kinase and polyclonal rabbit irisin and polyclonal rabbit beta-actin at a diluted concentration of 1:1,000. The signals were then visualized by anti-rabbit or anti-mouse horseradish peroxidase-conjugated secondary antibody (1:2,000). The results were visualized with Super Signal West Pico ECL chemiluminescence reagent (Thermo-Fisher Scientific). Densitometric analysis for the blots were completed using NIH Image J processing program.

### Statistical Analysis

Data were expressed as an average ± SEM of independent experiments. An unpaired, two-tailed Student t-test was used to determine significance between two groups. Multiple groups were analyzed using one-way ANOVA followed by Bonferroni post hoc test. Differences between groups were considered statistically significant when *p* < 0.05.

## Results

### PRAK Knockout Decrease Cell Viability When Exposed to High Glucose

**FIGURE 1:**
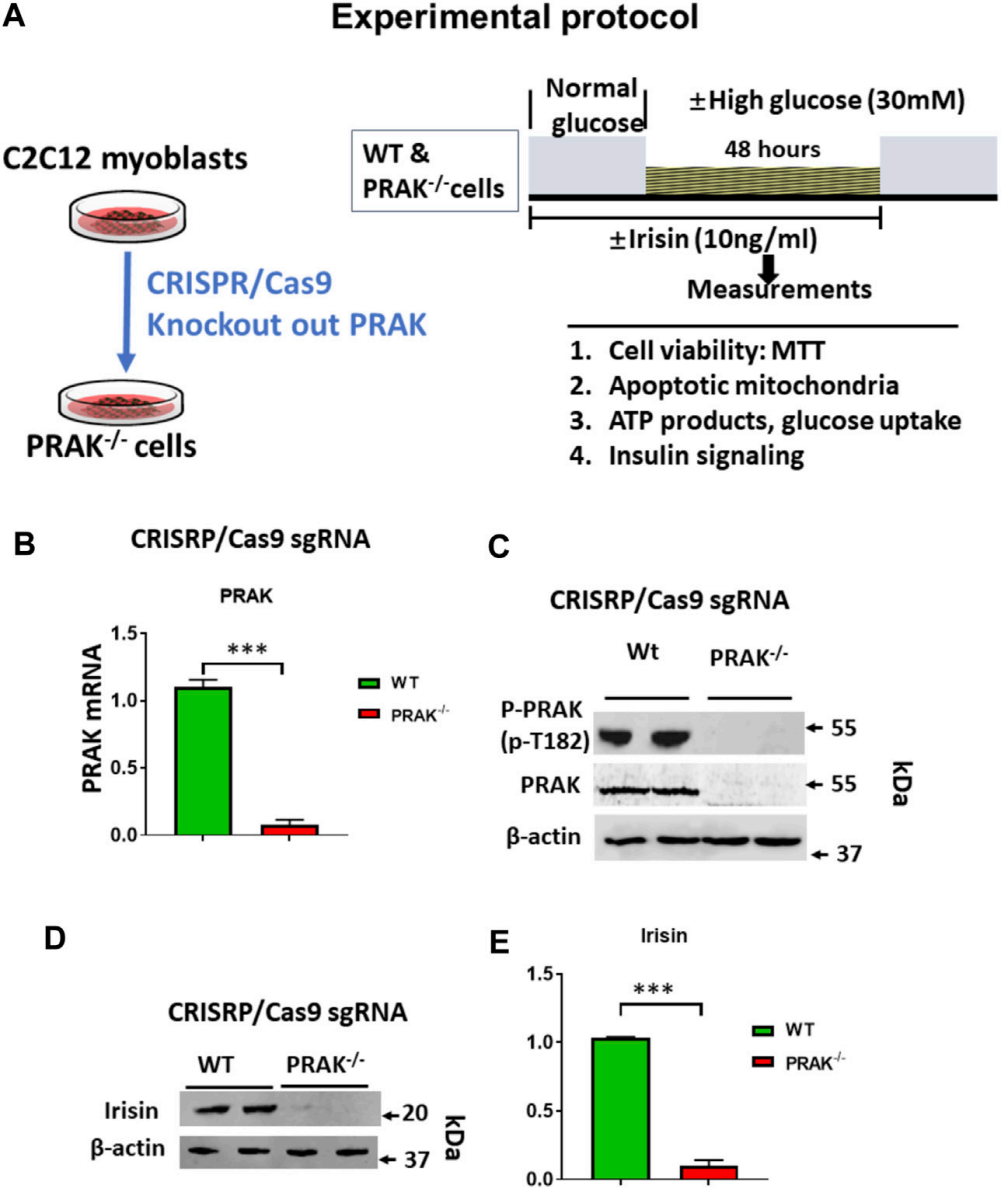
**(A)**: Experimental protocol: Generation of PRAK^−/−^ C2C12 cell lines using CRISPR/Cas 9 genome editing technology. CRISPR/Cas9 vector; sgRNA sequence; Myoblasts were differentiated to myotubes in which BSA medium was reduced from 10 to 2.5%, and C2C12 cells were cultured for 3 days to 1 week; high glucose (30 mM), normal glucose (5.6 mM) experimental protocol in C2C12 myoblasts. **(B)**: qPCR detected mRNA of PRAK in wild type and PRAK^−/−^C2C12 cells (*n* = 3/each group); Values represent means ± SEM (*n* = 3/group), ***p < 0.001. **(C)**: Western blot showed the absence of PRAK protein in PRAK^−/−^C2C12 cells; **(D)**: Western blot showed a reduction of irisin in PRAK^−/−^ cells; **(E)**: Densitometric analysis shows the reduction of irisin in PRAK^−/−^ cells; Values represent means ± SE (*n* = 5/each group), ***p < 0.001.

**FIGURE 2:**
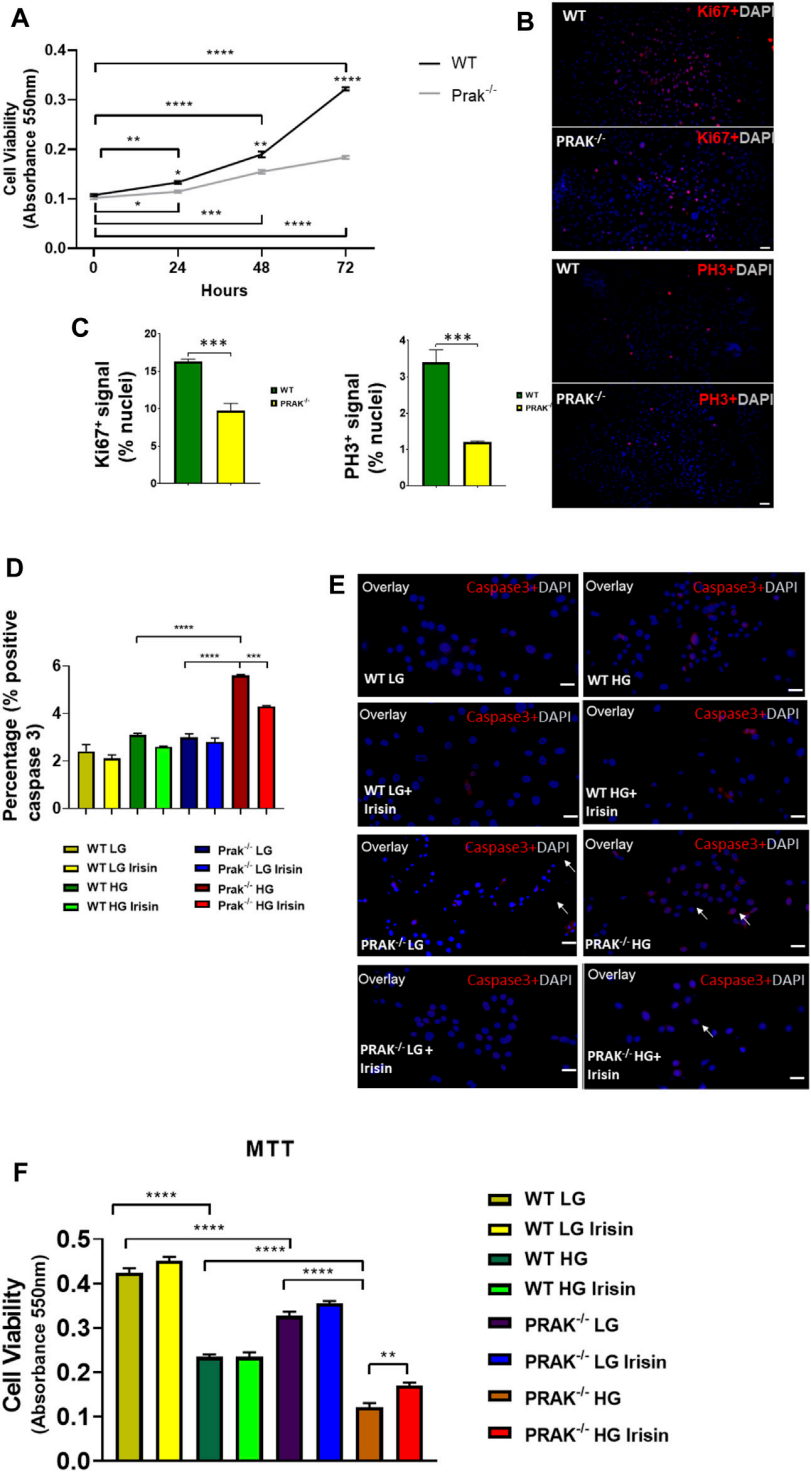
Knockout of PRAK suppresses cell growth and increased apoptosis of cells to high glucose damages: **(A)**: Knockout of PRAK delayed cell growth as estimated by 3-(4,5-dimethylthiazol-2-yl)-2,5-diphenyltetrazolium bromide (MTT) assay (*n* = 5/each group); *p < 0.05; **p < 0.01; ***p < 0.001, ****p < 0.0001. **(B)**: Immunochemical staining of Ki67 and phosphorylated histone 3 in both WT and PRAK^−/−^C2C12 cells; **(C)**: Quantification of Ki67 and phosphorylated histone 3-positive nuclei in wild type and PRAK^−/−^ C2C12 cells. **(D)**: Quantification of active caspase-3-positive nuclei among wild type and PRAK^−/−^ C2C12 cells in response to low glucose and high glucose with/without irisin treatment. Values represent means ± SE (*n* = 5/each group), there were approximately 50–100 cells were randomly accounted in each group; ***p < 0.001; ****p < 0.0001; active-caspase 3 was significantly reduced by irisin treatment. Irisin mitigated the increase in active caspase 3 in PRAK^−/−^ group. **(E)**: Representative images showing apoptotic C2C12 myoblasts in the low glucose condition: active caspase-3-positive nuclei in red (white arrows); nuclei were stained in blue (DAPI) **(left);** Representative images showing the apoptotic C2C12 myoblasts in the high glucose condition **(right):** active caspase-3-positive nuclei in red; nuclei were stained in blue (DAPI). Scale bar = 50 µm. **(F)**: PRAK knockout decreased cell survival (MTT) in myoblasts exposed to a high glucose (*n* = 5/each group). Values represent means ± SEM (*n* = 5/group). **p < 0.01; ****p < 0.0001.

The C2C12 were exposed to high glucose as shown in the experimental protocol (Figure 1A). As shown in Figure 1B, qPCR indicated that PRAK mRNA was remarkably reduced in PRAK^−/−^ in C2C12 myoblasts compared to wild type cells (Figure 1B). PRAK protein level was also absent in PRAK^−/−^ in C2C12 myoblasts (Figure 1C). We have established three PRAK^−/−^ clones, each of which demonstrated the absence of phosphorylated PRAK and non-phosphorylated PRAK proteins (Supplementary Figure S1). In addition, knockout of PRAK resulted in the reduction of irisin (Figures 1D,E). Deletion of PRAK induced slow growth compared to wild type cells (Figure 2A). Cell division was also suppressed as demonstrated by a lower Ki-67 labelling and phosphorylated histone 3 (PH3) signaling (Figures 2B,C). We subjected the cells to high glucose for the measurement of cellular apoptosis. As shown in Figure 2D, there was no significant active caspase 3 signaling in both wild type and PRAK^−/−^ cells at normal glucose levels. In response to high glucose stress, active caspase 3 positive cells were significantly increased relative to that of normal glucose levels. However, knockdown of PRAK elicited a greater elevation of active caspase-3 in cells exposed to high glucose, evident by a two-fold increase compared to wild type cells. Strikingly, administration of irisin resulted in a reduction of active caspase-3 positive signaling in wild type cells in response to high glucose. Likewise, PRAK^−/−^ cells treated with irisin demonstrated a marked reduction in apoptosis as compared to those with vehicle treatment, indicating that irisin rescued cells from the development of apoptosis induced by knockout of PRAK. Representative images are shown on Figure 2E in which red fluorophore indicates active caspase 3 positive cell where DAPI indicates nuclei. As shown in Figure 2F, wild type C2C12 myoblasts exposed to high glucose displayed cellular damage, indicated by the reduction of MTT compared with the normal glucose group. Likewise, Knockout of PRAK decrease affect cell viability rate in normal glucose levels but caused a remarkable reduction of MTT in response to high glucose concentration. In order to define whether irisin could rescue cells from high glucose-induced injury, both wild type and PRAK^−/−^ cells were treated with irisin at a concentration of (10 ng/ml) in response to high and normal glucose levels. Notably, irisin treatment rescued PRAK^−/−^ cells from high glucose-induced cellular injury, as evident by increased MTT.

### PRAK Knockout Decreased ATP and Glucose Uptake in Cells Exposed to High Glucose

**FIGURE 3:**
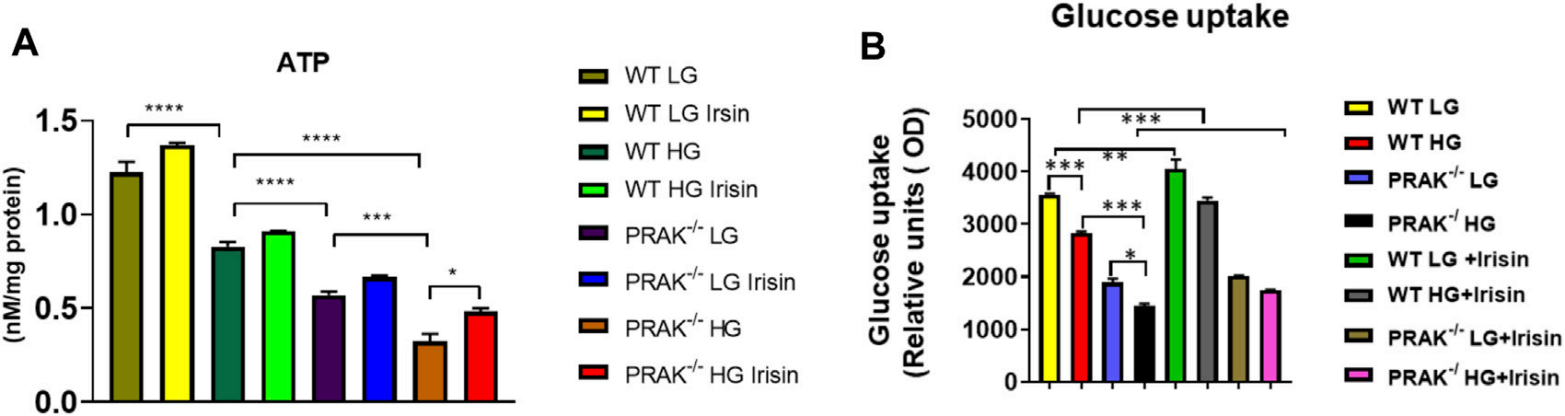
Deletion of PRAK reduced the ATP production and glucose uptakes of cells exposed to high glucose stimulus. **(A)** PRAK knockdown attenuated ATP content in cells exposed to high glucose. Mitochondria was isolated from C2C12 myoblasts, and the mitochondrial contents of ATP were measured (*n* = 3/each group). *p < 0.05; ***p < 0.001, ****p < 0.0001. Scale bar = 50 μm; **(B)** Intakes of fluorescent glucose analog 2-(N-(7-Nitrobenz-2-oxa-1,3-diazol-4-yl)Amino)-2-Deoxyglucose (2-NBDG) by PRAK and wild type cells were determined. Detailed culture condition is described in methods. Bar graph represents relative fluorescence uptake of 2-NBDG (*n* = 3/per group). *< 0.05; **< 0.01; ***p < 0.001.

As shown in Figure 3A, we have previously demonstrated that ATP synthesis was improved by irisin in c2c12 myoblasts exposed to high glucose (Yano et al., 2020). Knockout of PRAK decreases ATP synthesis even in the normal glucose condition. High glucose stress resulted in the further reduction of ATP synthesis in both wild C2C12 cells and PRAK^−/−^ C2C12 cells, but magnitude of ATP reduction in PRAK^−/−^ group was much more significant than wild type in high glucose treatment. Administration of irisin only marginally increased ATP production in wild type C2C12 cells. However, irisin administration increased ATP contents in PRAK^−/−^ C2C12 cells in high glucose treatment. In addition, As shown in Figure 3B, we measured the glucose uptake in both wild type and PRAK^−/−^ C2C12 cells, deletion of PRAK resulted in the decrease in glucose uptake even in low glucose concentration as compared to wild type cells. Glucose uptake was decreased in both PRAK^−/−^ and wild type cells exposed to 48 h of high glucose. However, the magnitude of glucose uptake reduction in PRAK^−/−^ cells was significantly larger than wild type cells, indicating that deletion of PRAK decreased glucose uptake in C2C12 exposed to high glucose. Treatment of cells with irisin enhanced the glucose uptake in both wild type cell and PRAK^−/−^ cells in either low glucose and high glucose treatment.

### PRAK Knockout Enhanced Mitochondrial Damage

**FIGURE 4:**
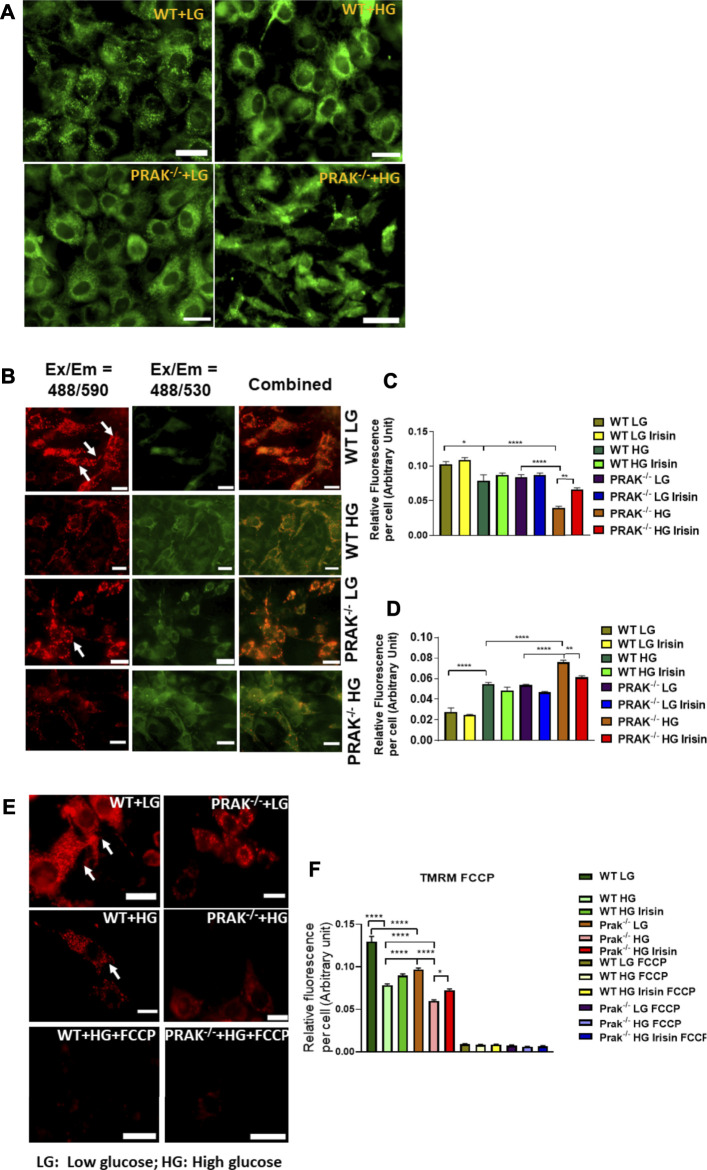
The effect of PRAK knockout on MMP reduction in C2C12 myoblasts exposed to high glucose. **(A)**: Mitochondrial tracker staining demonstrates the population of mitochondria in both wild type and PRAK^−/−^ C2C12 myoblasts. **(B–D)**; Myoblast mitochondrial damage was measured by assessing mitochondrial membrane depolarization. The MitoCapture dye that accumulates in healthy mitochondria emits a red signal, but MitoCapture diffuses into the cytoplasm and emits a green signal when mitochondria were subjected to damage. Exposing C2C12 myoblasts to high glucose resulted in a significant decrease in the ratio of red to green fluorescence intensity which indicates a sign of the early stages of cell apoptosis. Fluorescent intensities and representative images in red are shown in Figures 4B,C, respectively, **(B,D)**: Fluorescent intensities and representative images in green which indicate mitochondrial damage are shown in Figures 4B,D, respectively, mitochondrial injuries were more severe in the PRAK^−/−^ group. Irisin treatment significantly improved the high glucose-induced MMP loss in both WT and PRAK^−/−^ cells. Values represent means ± SE (*n* = 5/per group), *p < 0.05, **p < 0.01. ****p < 0.0001. **(E,F)**: Mitochondrial membrane potentials were evaluated using TMRM assay. TMRM is a positively charged and red-orange cell permeable dye that could readily accumulate in active mitochondria because of their relative negative charge. FCCP is an ionophore uncoupler of oxidative phosphorylation. FCCP treatment leads to depolarization, eliminates mitochondrial membrane potential, and suppresses TMRM staining as well. Representative images are shown in **(E)** TMRM, and bar graph represents mean relative fluorescence strength per cell **(F)** and, tetramethylrhodamine, methyl ester; FCCP, carbonilcyanide *p*-triflouromethoxyphenylhydrazone. Values represent means ± SE (*n* = 5/per group), *p < 0.05, ****p < 0.0001. Scale bar = 50 µm.

We used mitochondrial tracker to label living mitochondria in cells. As shown in Figure 4A, at a normal level of glucose, mitochondrial labeling demonstrates a healthy status and no difference between wild type and PRAK^−/−^ cells. When PRAK^−/−^ cells were subjected to a high glucose level, both populations of living mitochondria did not change significantly in population in response to the high glucose stimulus. The mitochondrial membrane potential (MMP) acts as an early event in development of mitochondrial apoptosis. To determine the effect of PRAK on the state of MMP, MitoCapture, a cationic dye in living cells, was used to evaluate apoptosis in each condition. MitoCapture accumulates in mitochondria under the condition that mitochondrial function is intact and emits a red signal and is in apoptotic mitochondria the signal becomes green. As shown in Figure 4B representative image and Figures 4C,D, as compared to the normal glucose condition, C2C12 exposed to a high glucose level lost the red fluorescent signals but increased green signals as compared to the normal glucose levels which was further exacerbated in PRAK^−/−^cells. There was observable fluorescent signal between wild type and PRAK^−/−^cells at a normal glucose level. However, irisin treatment showed a protective role in PRAK^−/−^ cells and inhibited loss of MMP. The MMP was further assessed by utilizing the red-fluorescent probe TMRM (tetramethylrhodamine, methyl ester), which is localizing to mitochondria and used for the detection of mitochondrial membrane depolarization. As illustrated in Figures 4E,F, measurements of the intensity of TMRM in all groups showed patterns similar to MitoCapture measurements. Notably, treatment with FCCP, an uncoupler of mitochondrial oxidative phosphorylation, induced cells to display the minimum intensity of fluorescence. The data suggest that knockout of PRAK augmented mitochondrial damage as evident by loss of mitochondrial membrane potential in response to high glucose stress, which was rescued by administration of irisin.

### PRAK Deletion Decreases, PI3 Kinase, IRS-1, and AMPKα Phosphorylations

**FIGURE 5:**
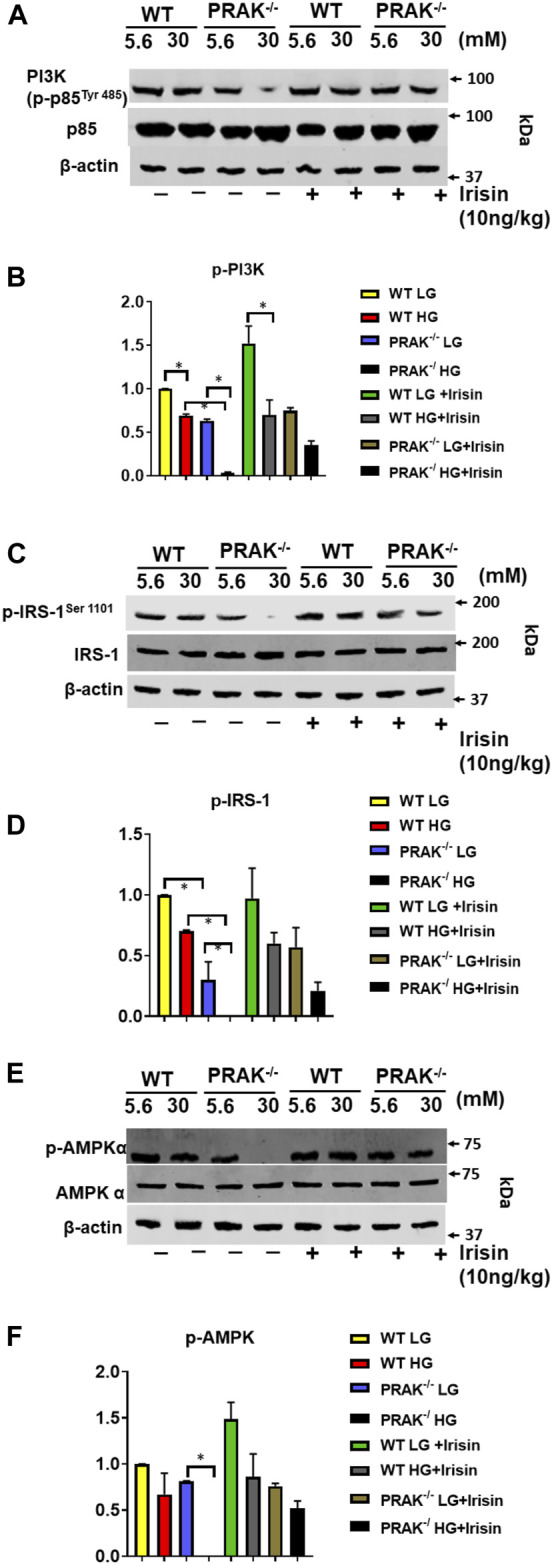
Deletion of PRAK suppresses insulin signaling in C2C12 myoblasts exposed to high glucose. **(A)** PRAK^−/−^ and wild type cells were cultured under the indicated conditions for 48 h in either normal glucose or high glucose concentrations. Cell lysates underwent western blotting using anti phospho (*p*) and total (t), PI3 Kinase (p85 at Tyr 485) **(A,B)**, or IRS-1 **(C,D)** AMPKα **(E,F)** antibodies. **(A)** Representative blot of PI3 Kinase (p85 at Tyr 485) and non-phosphorylated PI3 Kinase; **(B)** Bar graph **(lower panel)** represents ratios of PI3 Kinase (p85 at Tyr 485) against non-phosphorylated PI3 Kinase from densitometric analysis (*n* = 3/per group); **(C)**: Representative blot of phosphorylated IRS-1 and non-phosphorylated IRS-1; **(D)** Bar graph** (lower panel)** represents ratios of phosphorylated IRS-1 against non-phosphorylated IRS-1 from densitometric analysis (*n* = 3/per group); **(E)** Representative blot of phosphorylated AMPKα and non-phosphorylated AMPKα; Bar graph **(lower panel) **represents ratios of phosphorylated AMPKα against non-phosphorylated AMPKα from densitometric analysis. Values represent means ± SE (*n* = 3 samples/per group). *p < 0.05.

Assessment of phosphorylation status of downstream targets of insulin receptor provides insight into insulin signaling pathway activation. As shown in Figures 5A,B, phosphorylated PI3 kinase (p85 at Tyr 485) decreased by deletion of PRAK as compared to wild type C2C12 cells. High glucose caused the further reduction of phosphorylation of phosphorylated PI3 kinase, whereas non-PI3 kinase was not changed by either deletion of PRAK or high glucose. Administration of irisin resulted in an increase in phosphorylation of PI3K in PRAK^−/−^ C2C12 in high glucose whereas there was no difference in non-phosphorylated PI3K kinase among the group. Likewise, as shown in Figures 5C,D, phosphorylation of IRS-1 was suppressed following knockdown of PRAK as compared to wild type cells although there was no significant different; there was no difference in non-phosphorylated IRS-1 *p* in PRAK^−/−^ cells. PRAK knockout resulted in almost undetectable phosphorylated IRS-1 as compared to wild type cells in response to high glucose. However, PRAK knockdown-induced inhibition of IRS-1 phosphorylation was rescued following irisin treatment, indicating that irisin downstream of PRAK could activate the IRS-1 downstream pathway or independently of PRAK. As shown in Figures 5E,F, AMP phosphorylation was detected in both wild type and PRAK^−/−^ cells in the normal condition without notable differences. High glucose levels caused a mild loss of the phosphorylation of AMPK in wild type cells, but AMPK phosphorylation was not detectable by the knockdown of PRAK. Irisin has been the focus of much researches in terms of its relationship with AMPK (Gwinn et al., 2008; Park et al., 2015). Interestingly, irisin treatment led to a marked augmentation in AMPK phosphorylation in both wild type and PRAK^−/−^ cells. Notably, knockout of PRAK induced an abatement in AMPK phosphorylation that was rescued by irisin treatment in response to high glucose stress.

## Discussion

Salient findings: In this study, we used CRISPR/Cas9 genome editing technology to knockdown PRAK. Our results demonstrated that knockdown of PRAK decreased the viability of C2C12 myoblasts to high glucose treatment. Knockout of PRAK reduced ATP synthesis and glucose uptakes in C2C12 myoblasts exposed to high glucose stress. Also, high glucose stress resulted in an increase in apoptosis, which was further elevated by knockout of PRAK. Mitochondrial membrane potentials were suppressed by knockout of PRAK, which was exacerbated in response to uncoupling of mitochondrial oxidative phosphorylation. High glucose caused an impairment in insulin signaling as evident by reductions in phosphorylated PI3K kinase (p85 at Tyr 485), insulin receptor-1 and AMPK. Notably, irisin, a newly identified hormone, remarkably rescued PRAK knockout-induced decrease in cell viability and impairment of mitochondrial function from high glucose stress. Our results indicate that PRAK plays a critical role in modulating mitochondria function, glucose uptake and insulin signaling in C2C12 cells exposed to high glucose, which his highly related to irisin.

Zheng et al. demonstrated that a specific p38 kinase cascade/PRAK plays a critical role for energy depletion induced inhibition of mTORC1 (Sabatini et al., 1994; Loewith et al., 2002; Jacinto et al., 2004; Zheng et al., 2011). Energy depletion activates AMPK, which is closely related to modulating the phosphorylation of mTOR1 regulatory associated protein (Garami et al., 2003; Inoki et al., 2003; Gwinn et al., 2008; Hardie, 2008). Our results indicate that knockout of PRAK aggravated the reduction of AMPK phosphorylation in response to high glucose stress. It is not yet clear whether PRAK-knockout induced reduction was due to stimulation of the mTOR pathway. We have found that deletion of PRAK resulted in decreases in PI3 kinase (p85 at Tyr 485), IRS-1 and AMPK phosphorylation as compared to wildtype control. However, the inhibitory effect of PRAK knockout on PI3 kinase (p85 at Tyr 485), AMPK and IRS-1 phosphorylation was prevented by irisin, suggesting that irisin may act as one of the downstream targets of PRAK to modulate PI3 kinase (p85 at Tyr 485), AMPK and IRS-1. Obesity and insulin resistance were promoted in response to stress, which is in part mediated by stimulation of MAPKs, including p38 MAPK or induction of the expression of MAPK phosphatase-1 (MKP-1). Mice lacking MKP-1 (MKP1-MKO) in skeletal muscle demonstrated increased skeletal muscle p38 MAPK, which manifested in resistance to development of obesity in mice exposed to diet intervention (Lawan et al., 2018). Another observation shows that mice lacking p38γ/δ in myeloid cells displayed resistance to the development of diet‐induced fatty liver and glucose intolerance, which contributes to p38 deficiency in neutrophil infiltration, thereby promoting the development of steatosis and liver metabolic changes (González-Terán et al., 2016).

We also found that knockout of PRAK reduced cell viability in response to a high glucose stress, which is in line with reports showing that ablation of PRAK enhanced myocardial ischemia and reperfusion injury and promoted myocardial remodeling (Zhao et al., 2019). Mitochondrial dysfunction occurred in response to high glucose stress, which was further exacerbated by deletion of PRAK, indicating that PRAK is required for preserving mitochondrial function in high glucose stress. This is supported by another study in which activation of p38 during exercise resulted in greater phosphorylated and activated PGC-1α, which translocated into the nucleus as well as co-activating transcriptional factors and nuclear receptors, leading to expression of mitochondrial proteins (Wright et al., 2007). p38 MAPK is identified to directly phosphorylate the repressor regions of PGC-1α and leads to a protein that is both more transcriptively active and more stable (Puigserver et al., 2001; Teyssier et al., 2005).

It was reported that p38 is upstream of PGC-1α, resulting in the stimulation of FNDC5. A cleavage product of the extracellular portion of FNDC5 is secreted and circulated into peripheral circulation to act as a myokine (Boström et al., 2012; Park et al., 2015; Zhu et al., 2015). It is not yet clear if PRAK serves as an upstream signal of irisin and could rescue cells from high glucose stress-induced cellular death. Interestingly, PRAK deletion-induced reduction in cell viability were rescued by irisin, indicating that irisin acts as a downstream signaling pathway. This is in agreement with studies establishing that p38 plays a key role in mediating the production of PGC-1 (Cao et al., 2004; Sanchis-Gomar and Perez-Quilis, 2014; Wu and Spiegelman, 2014; Zhang et al., 2014; Zhang et al., 2017). In addition, deletion of PRAK induced-increases in mPTP and mitochondrial apoptosis were mitigated by irisin treatment, indicating the irisin could rescue the mitochondria damage in PRAK^−/−^ C2C12 myoblasts. High glucose stress reduced the synthesis of ATP production in cells exposed to high glucose, the extent to which was further attenuated by knockout of PRAK. Reduction of ATP following PRAK deletion may be related to the suppression of PGC-1α. Provision of irisin recovered ATP contents in cells exposed to high glucose. Reduction of ATP in PRAK^−/−^ C2C12 myoblasts is likely to result from impairment in mitochondrial function by deletion of PRAK. Furthermore, we have found that deletion of PRAK resulted in an impairment in glucose uptake as compared to wild type cells, where irisin treatment induced the increase in glucose uptake even in PRAK^−/−^ C2C12 cells, suggesting the key role of PRAK in mediating glucose uptake and that irisin could be downstream of PRAK in regulation of glucose uptake. The detrimental effect of PRAK knockout in C2C12 cells was rescued by irisin, indicating a critical correlation between PRAK and irisin, but molecular regulation by which how PRAK directly interacts with and modulates irisin is still unclear at the present, which needs further investigation. In experimental diabetes, muscles with a predominantly slow-twitch fiber profile exhibit greater insulin sensitivity and greater glucose uptake than muscles with a predominantly fast-twitch fiber profile (Snow and Thompson, 2009; Takemura and Ishihara, 2016). We have found that glucose uptake decreased by deletion of PRAK in normal and high glucose exposures. This is likely to be associated with the decreased activity of insulin signaling such as IRS-1, PI3K and AMPKα. In addition, glucose uptake was preserved by treatment of irisin, which is in agreement with our previous observation that irisin plays critical role in promoting glucose uptake (Yano et al., 2020). We have found that knockout of PRAK attenuated the proliferative rate of C2C12 cells. We did not examine whether knockout of PRAK could also affect the phenotype of differentiated C2C12 cells in response to a differentiation condition. Given that p38, an upstream target of PRAK, plays a critical function in modulating cell differentiation, it is possible that knockout of PRAK could also affect the differentiation of cells, which merits further investigation. In our study, we demonstrated that irisin acts as a potential downstream target of PRAK and rescued the detrimental effect of PRAK deletion. It will be interesting to see whether the re-introduction of PRAK gene in PRAK^−/−^ C2C12 could also rescue the effects of PRAK knockout, which could provide an interesting line to further confirm the effects of PRAK. Also, there is a limitation in this study in which only an *in vitro* model was implemented to investigate the correlation between PRAK, irisin, and insulin signaling, a future experiment will also be important to define if this unique pathway also plays an role in regulation of insulin signaling pathway using *in vivo* model, which could provide clinically relevant evidence for design therapeutic strategy.

## Conclusion

**FIGURE 6:**
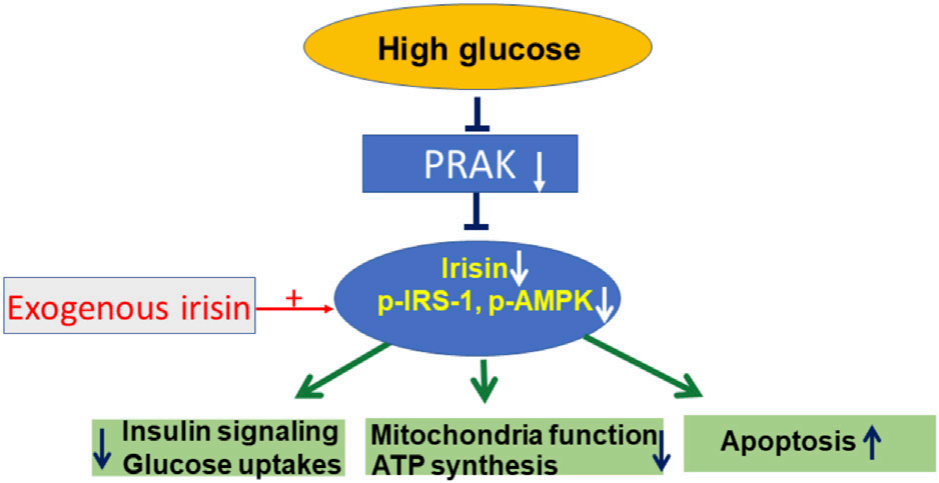
The central mechanism of PRAK and irisin in regulation of insulin signaling and mitochondria function.

PRAK presents a novel molecular target that closely link irisin to the regulation of mitochondrial function, ATP production and glucose uptake. Knockout of PRAK reduces cell viability in response to high glucose stress, which was mitigated by irisin. Furthermore, deletion of PRAK suppressed phosphorylation of PI3 kinase, IRS-1, and AMPKα in cells exposed to high glucose stress, which was prevented by irisin treatment. The mechanism of RPAK in regulation of high glucose-induced cellular signaling is summarized in Figure 6, which was rescued by treatment of exogenous irisin. Taken together, the studies presented here suggest an important role for PRAK in modulating mitochondrial function, glucose uptake and insulin signaling in response to a high glucose stress.

## Data Availability

The raw data supporting the conclusions of this article will be made available by the authors, without undue reservation.
