# Supplementary figures and images for: Deletion of PRAK Mitigates the Mitochondria Function and Suppresses Insulin Signaling in C2C12 Myoblasts Exposed to High Glucose
Source: Front Pharmacol. 2021 Oct 4;12:698714. doi: 10.3389/fphar.2021.698714 (PMC8521062; doi:10.3389/fphar.2021.698714)

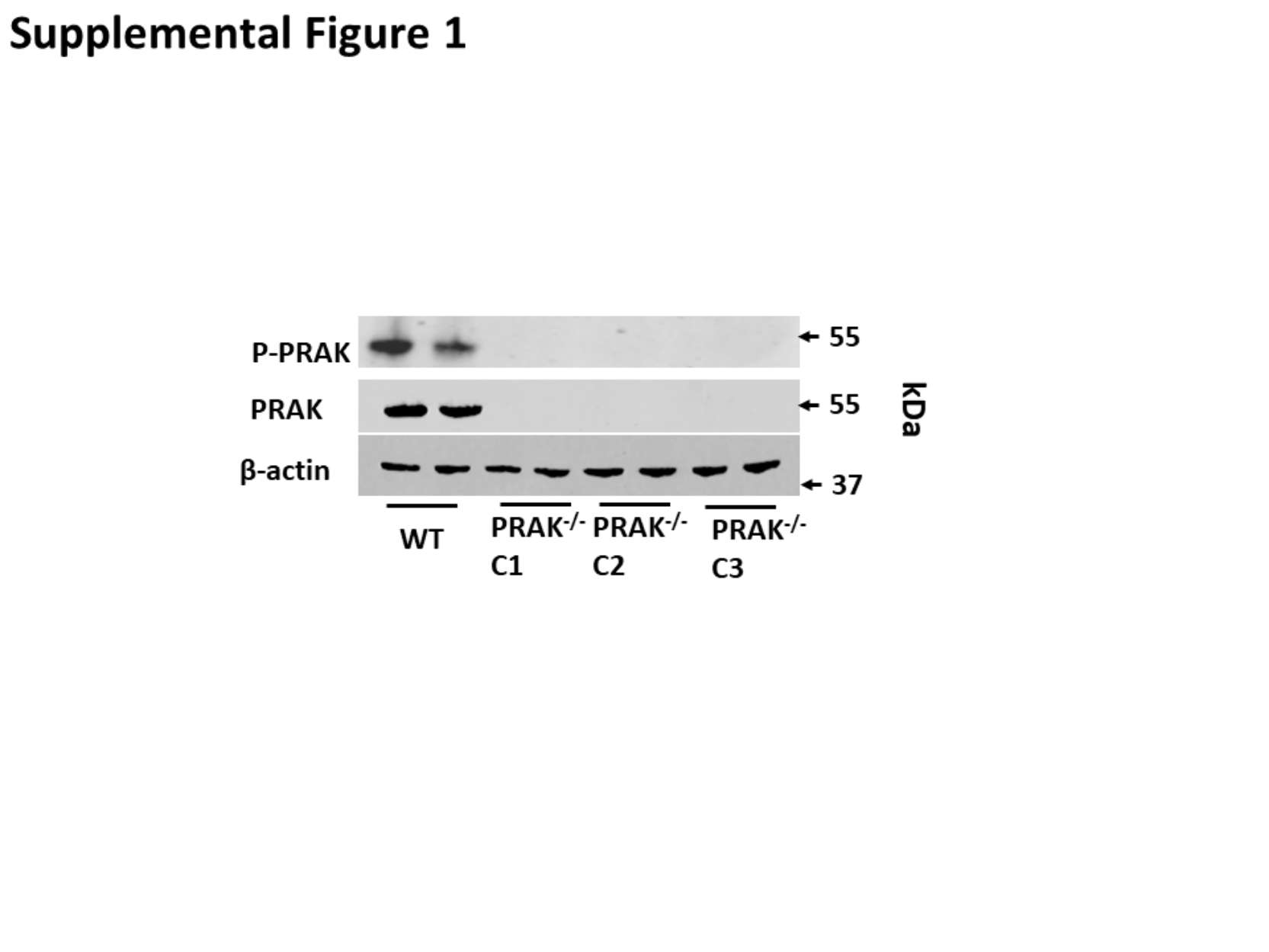

Supplement: Supplementary file 1 [file Image1.TIF]
